# Effects of Contact Angle and Flocculation of Particles of Oligomer of Tetrafluoroethylene on Oil Foaming

**DOI:** 10.3389/fchem.2018.00435

**Published:** 2018-09-24

**Authors:** Ryo Murakami, Syuji Kobayashi, Manami Okazaki, Alexander Bismarck, Masahiro Yamamoto

**Affiliations:** ^1^Department of Chemistry, Konan University, Kobe, Japan; ^2^Polymer and Composite Engineering (PaCE) Group, Department of Material Chemistry, University of Vienna, Vienna, Austria; ^3^Polymer and Composite Engineering (PaCE) Group, Department of Chemical Engineering, Imperial College London, London, United Kingdom

**Keywords:** oil foams, Pickering-Ramsden foams, contact angle, adsorption, flocculation

## Abstract

Oil foams have been stabilized by using particles of oligomer of tetrafluoroethylene (OTFE). OTFE particles were dispersed in oil mixtures prior to aeration, to exclude the oil-repellency nature of the particles due to the formation of the metastable Cassie-Baxter state and properly evaluate the effects of contact angle on the foaming behavior. The particle contact angle (θ^Y^) against air/oil surfaces were controlled by changing a composition of two oils with different surface tension (*n*-heptane and methyl salicylate). The θ^Y^ value increases with increasing a mole fraction of methyl salicylate, from 42° (for pure *n*-heptane) to 89° (for pure methyl salicylate). The air volume incorporated in the oils after aerating OTFE dispersions in the oil mixtures shows a maximum when θ^Y^ = 55°. The flocculation of OTFE particles in bulk oils is responsible for the unexpected behavior of foaming observed when θ^Y^ is relatively high. The increase in the degree of the flocculation reduces the effective concentration of OTFE particles in bulk oil, leading to the inefficient bubble stabilization. These findings suggest the efficient oil foaming using particles as a stabilizer is achieved by optimizing both the particle contact angle and the degree of flocculation in oils.

## Introduction

Liquid foams are utilized in industries such as foods and cosmetics but also in the chemical processes such as froth flotation and foam fractionation. Molecular and polymeric surfactants have been frequently used to stabilize liquid foams. Particulate materials (colloidal particles) are also adsorbed at air/liquid surfaces, usually forming adsorbed films of densely packed particles, which prevents coalescence and disproportionation, as has been reported for particle-stabilized emulsions (Pickering-Ramsden emulsions) (Binks and Horozov, 2006). Water is the most frequently used liquid for foaming with particulate materials, there are a large of number studies on particle-stabilized aqueous foams (Alargova et al., 2004; Binks and Horozov, 2005; Binks and Murakami, 2006; Gonzenbach et al., 2006; Kostakis et al., 2006; Binks et al., 2007; Horozov, 2008; Wege et al., 2008; Stocco et al., 2011). Non-aqueous liquids that are practically not miscible with water and show a relatively low surface tension, that is, oils can be also aerated in the presence of particles (Friberg, 2010; Fameau, 2017). One of the important factors for stabilizing foams and emulsions with particles is the particle wettability against air/liquid surfaces, quantified by a contact angle (θ). For dispersed systems consisting of air and liquids, if a particle is relatively liquid-philic, air-in-liquid systems (liquid foams) are stabilized, while relatively liquid-phobic particles stabilize liquid-in-air systems, such as liquid marbles and dry liquids. The phase inversion between the two systems has been achieved by controlling the particle wettability (Binks and Murakami, 2006; Binks et al., 2007; Murakami and Bismarck, 2010).

The driving force of adsorption of a particle to an air/liquid surface is the free energy gain (Δ*G*) by losing an area of the surface and crucially dependent on θ (Binks and Horozov, 2006). If a particle is spherical and the line tension effect is neglected, Δ*G* can be calculated as an energy to remove the particle already adsorbed at a liquid surface into bulk;

(1) $$\begin{array}{l} \Delta G=\pi r^{2}\gamma_{\text{al}}{\left(1-|\text{cos }\theta |\right)}^{2}. \end{array}$$

Here *r* is a particle radius, γ_al_ is a surface tension between air and liquid. For dispersed systems of air and liquids θ is measured through the liquid phase. At fixed surface tension and particle size, Δ*G* shows a maximum at 90° and sharply decreases with θ either decreasing to 0° or increasing to 180°. The Δ*G* for the particles with moderate wettability is typically higher than hundreds of *kT*, implying such particles are irreversibly adsorbed at fluid interfaces. This irreversible adsorption has been thought to be the origin of the stability of particle-stabilized dispersed systems. Aqueous foams stabilized solely by nano-sized silica particles with different hydrophobicity have been prepared (Binks and Horozov, 2005; Binks and Murakami, 2006; Binks et al., 2007). When the hydrophobicity is intermediate, stable aqueous foams are formed and the foaming efficiency increases with increasing the hydrophobicity. If the particles are very hydrophilic, that is, θ is relatively close to 0°, the particles are easily desorbed from an air/water surface and prefer remaining in a water phase, leading to the formation of particle dispersions.

Compared to particle-stabilized aqueous foams, particle-stabilized oil foams have been sparsely studied (Friberg, 2010; Fameau, 2017). The stabilization of oil foams have been reported using particles with low surface energy (particles with fluorinated surfaces and fluoro-particles such as PTFE) and various kinds of pure oils (Thareja et al., 2008; Binks et al., 2011, 2015; Binks and Tyowua, 2013). The types of materials formed by mixing air, the oils and the particles have been considered in terms of air-oil surface tension, particle surface energy and mixing methods. However, the mechanisms regarding oil foam stabilization, for example, the dependence of foam volume on the contact angle, has not been studied. In our previous study, we have prepared oil foams using oligomer of tetrafluoroethylene (OTFE) particle (Murakami and Bismarck, 2010). The particle contact angle was controlled by mixing two oils with different surface tension. The foam volume shows a maximum when the particle intrinsic (Young's) contact angle (θ^Y^), which is the contact angle determined by the particle surface chemistry, is around 46°. When the contact angle is relatively high, say, between 50 and 80°, oil foams are barely formed, instead, agglomerated particles containing small amount of trapped air bubbles are obtained. From the viewpoint of Δ*G*, the foaming efficiency is expected to increase with increasing θ^Y^ when θ^Y^ < 90°, as is found for particle-stabilized aqueous foams (Binks and Horozov, 2005; Binks and Murakami, 2006). The reason of the poor oil foaming at a relatively high θ^Y^ is that OTFE particles are oil-repellent due to the metastable Cassie-Baxter state. The particles are highly agglomerated in air due to the surface force between the particles and air/oil surfaces can be suspended between the primary particles in the agglomerates, leading to the formation of the metastable Cassie-Baxter state, when θ^Y^ is higher than a critical angle (46°). Bubbles formed during aeration are barely adsorbed by OTFE particles, as they are not readily wetted by oils when θ^Y^ is higher than 46°. Such oil-repellent agglomerated particles preferably stabilize oil marbles and dry oils, instead of oil foams.

We have hypothesized that if OTFE particles are properly dispersed in oil *prior to aeration*, oil foam volume would increase with increasing θ^Y^ and hence with increasing Δ*G*. In this study, we prepare dispersions of OTFE particles in two oils with a low surface tension oil (*n*-heptane) and a high surface tension one (methyl salicylate). To eliminate the effects of the metastable Cassie-Baxter state on oil foaming behavior, a dispersion of OTFE particles in methyl salicylate is prepared by using the solvent replacement method; initially the particles are dispersed in an oil with a low surface tension (hence low θ^Y^) and the oil is gradually replaced with an oil with a high surface tension (hence high θ^Y^). The two dispersions of OTFE particles are mixed at a fixed content of OTFE particles and θ^Y^ is increased with increasing a mole fraction of methyl salicylate in the oil mixture. By aerating the dispersions of OTFE particles with showing different θ^Y^, we investigate the dependence of oil foaming on the contact angle and discuss the oil foaming mechanism. The aim of this study is to find out important factors to control properties of oil foams stabilized by particulate materials.

## Experimental

### Materials

**Figure 1:**
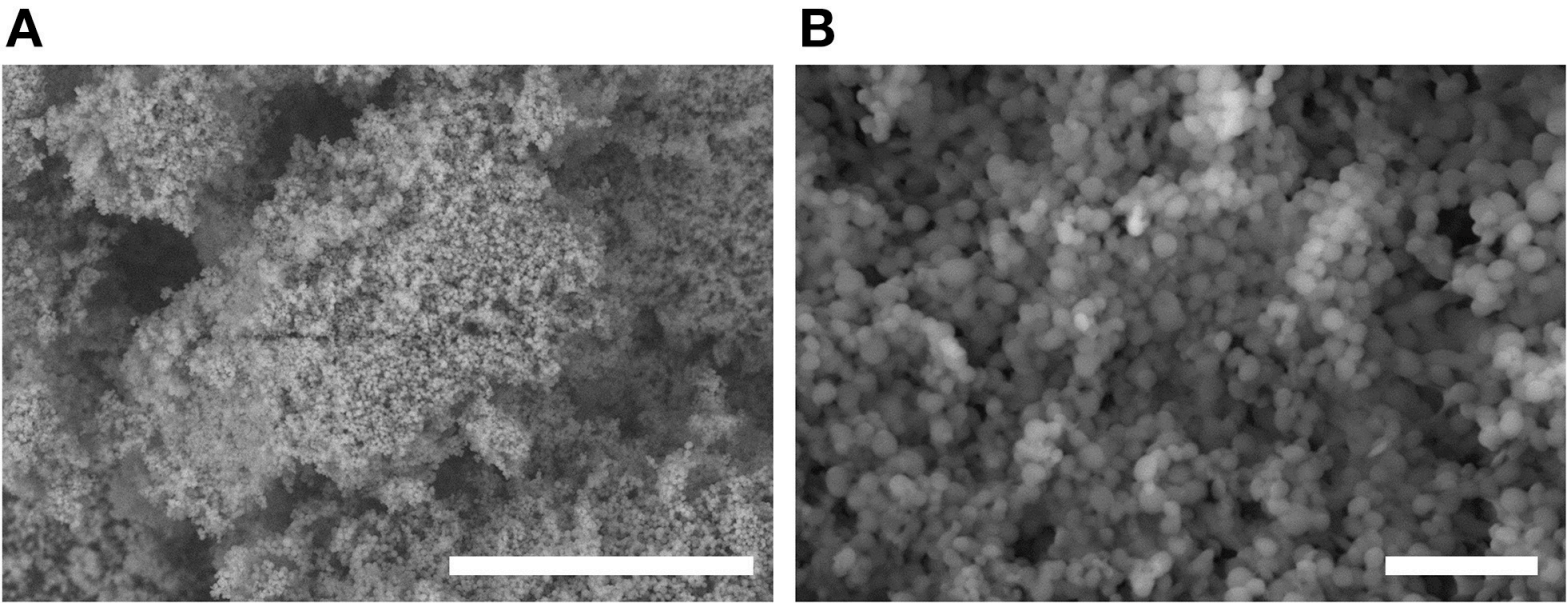
Variable pressure-SEM images of OTFE particles taken at **(A)** lower and **(B)** higher magnification. Scale bar = 50 μm for **(A)** and = 5 μm for **(B)**.

OTFE particles (surface area = 2.17–3.26 m^2^ g^−1^, supplier information) were supplied by Central Glass Co. Ltd., Japan. The particle density is 2.3 g cm^−3^, but the bulk density is 0.30 g cm^−3^, indicating that the powder contains about 90 vol.% air. The degree of polymerization is 5 to 145, corresponding to the molecular weight of 700 to 4,000. Scanning electron microscope images of OTFE particles without any conductive coating are shown in Figure 1. The primary particles are nearly spherical with diameters of 0.3 to 1.4 μm (Figure 1B). Each primary particle is strongly agglomerated in air due to surface forces. The size of the particle agglomerates is tens to hundreds of micrometers (Figure 1A).

Two oils were used. The one is an oil with a low surface tension, *n*-heptane [γ = 19.66 mN m^−1^ at 25°C (Lide, 2006)], and the other is an oil with a high surface tension [39.2 mN m^−1^ at 25°C (Lide, 2006)], methyl salicylate. Methyl salicylate, *n*-heptane (SAJ special grade, ≥ 99.0%, Sigma-Aldrich Japan) were passed through alumina columns twice prior to use.

### Contact angle measurement

To estimate a contact angle of OTFE particles against air/oil surfaces (θ^Y^), compressed tablets of OTFE particles were prepared by pressing 0.21 g of the particles in evacuable pellet dies (diameter 13 mm, Specac) at 81.2 MPa for 30 min. Contact angle measurements were conducted using a LSE-B100 (NiCK Corp., Japan) at room temperature (22 ± 2°C). About 10 μL of mixtures of the two oils was placed on the tablet and then advancing contact angles were measured. The error of the measurement is typically < 2°. The compressed tablets are reasonably smooth, judging the difference between advancing and receding contact angles, i.e. the contact angle hysteresis of oils on the tablets is smaller than 3°.

### Preparation of particle dispersions in oil mixtures and non-aqueous foams

Two dispersions of OTFE particles in different oils were prepared. The dispersion of OTFE particles in *n*-heptane was prepared simply by contacting OTFE particles with the oil, as the particles were spontaneously wetted by the oil. The dispersion of OTFE particles in methyl salicylate was prepared by the following several steps (solvent replacement method). Firstly, an amount of OTFE dispersion in *n*-heptane was centrifuged. Secondly, the supernatant brought about by the centrifugation was removed and pure methyl salicylate was added on top of the sedimented OTFE particles. OTFE particles were then re-dispersed by sonication and sedimented again by centrifugation. By repeating these re-dispersion and sedimentation several times, *n*-heptane was practically completely removed and an OTFE dispersion in methyl salicylate was obtained; the final *n*-heptane concentration in OTFE dispersion in methyl salicylate was < 0.01 wt%.

By mixing the OTFE dispersions in *n*-heptane and methyl salicylate, we have prepared OTFE dispersions with desired mole fractions of methyl salicylate (*x*_MS_) and particle concentrations (1.0, 2.0 and 3.0 vol.% relative to the total volume). The liquid volume was fixed at 5.0 mL. Aeration of OTFE dispersions in glass vials with a volumetric capacity of about 20 mL was carried out by shaking the dispersions by hand at 4 Hz for 15 s, at room temperature (22 ± 2°C).

### Characterization

The air volume incorporated in oil mixtures was measured manually at a certain time. The error for the volume measurement is typically ± 0.1 mL. Photographs of samples were taken with a CX2 digital camera (Ricoh). Optical micrographs of samples placed on a glass slide with a depression were taken with an Olympus BX51 transmission/reflection microscope fitted with a CMOS camera (Moticam 2000, Shimazu). SEM observation without prior conductive coating was conducted using a Hitachi S-3400N Variable Pressure SEM at an accelerating voltage of 15 kV and at 60-80 Pa with an environmental secondary electron detector.

## Results and discussion

### Contact angle

**Figure 2:**
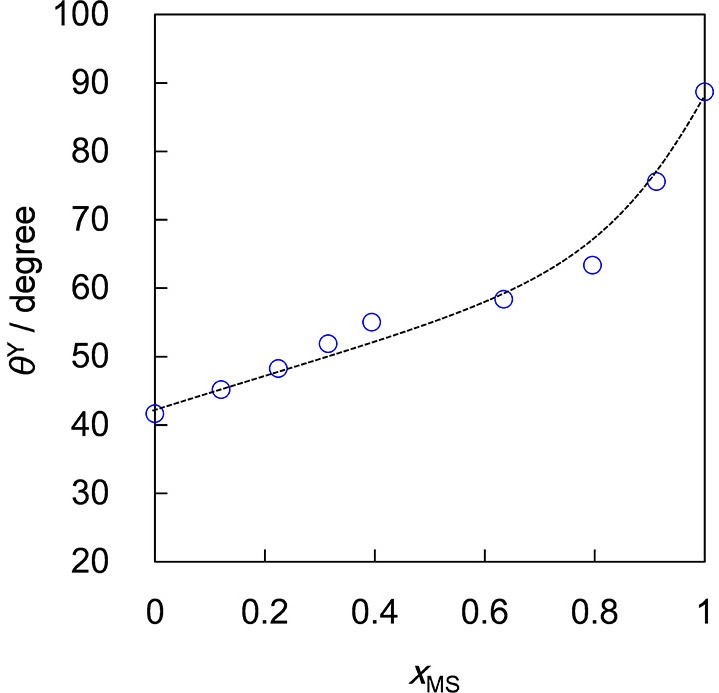
Contact angle of OTFE particles against air/oil surface (θ^Y^) as a function of mole fraction of methyl salicylate (*x*_MS_).

It is challenging to measure the contact angle of a small particle adsorbed at a liquid surface (Binks et al., 2011). Instead we have measured contact angles of oil drops on tablets of OTFE particles prepared by compression molding, which could mimic the particle surface (Murakami and Bismarck, 2010). The θ^Y^ value measured through an oil phase monotonically increases from 42° (for pure *n*-heptane) to 89° (for pure methyl salicylate) with increasing *x*_MS_, as shown in Figure 2. The θ^Y^ value for pure *n*-heptane (42°) is lower than the critical contact angle for the formation of the metastable Cassie-Baxter transition (46°). In fact, a dispersion of OTFE particles in *n*-heptane was obtained by simply placing the particles on the top of the surface of the oil. On the other hand, OTFE particles repels the oil mixture when *x*_MS_ > 0.22 (θ^Y^ > 48°) due to the formation of the metastable Cassie-Baxter state (Murakami and Bismarck, 2010). To properly disperse OTFE particles and study the effect of the contact angle on the foaming behavior in the whole range of *x*_MS_, the solvent replacement method was used.

### Oil foaming behavior

**Figure 3:**
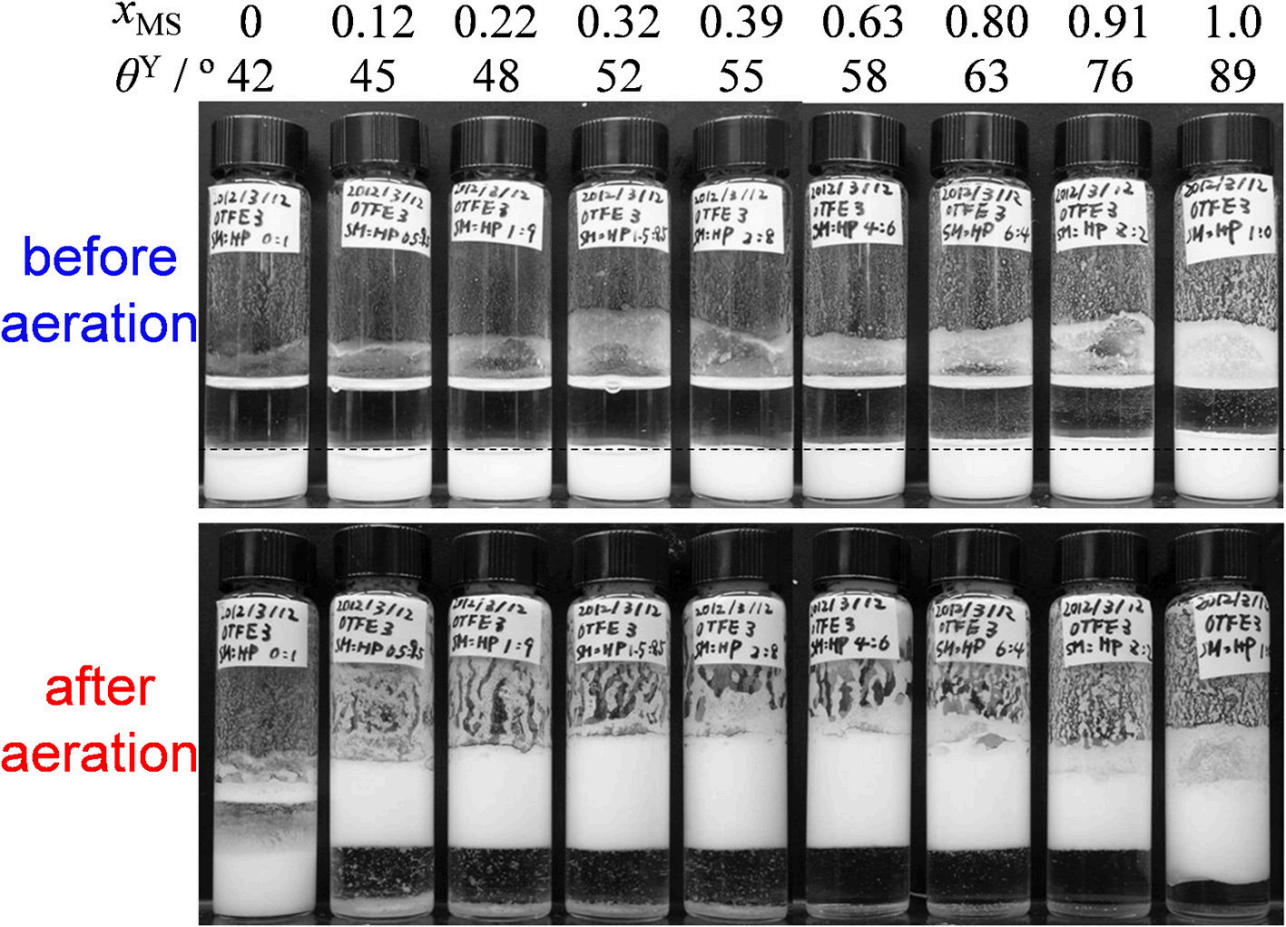
Digital photographs of vials containing 3.0 vol.% dispersions of OTFE particles in oil mixtures at different *x*_MS_ (and θ^Y^) before and after aeration. Dotted line is guide for apparent volume of sedimented OTFE particles.

To remove the oil-repellent character and evaluate effects of contact angle on oil foaming, we have dispersed OTFE into oils by the solvent replacement. The upper part of Figure 3 shows a digital photographs of vials containing 3.0 vol.% dispersions of OTFE particles in oil mixtures at different *x*_MS_ (and θ^Y^) before aeration. All the OTFE dispersions with different *x*_MS_ (θ^Y^) show the same total volume, showing OTFE particles are completely wetted by the oil mixtures and they are properly dispersed in the oils. The particles sediment on the bottom of the glass vials, as the particle density is much higher than the oil one. It should be noted that the apparent volume of the sedimented particles increases with increasing θ^Y^, when θ^Y^ ≧ 55°, while the volume is identical when 42° ≦ θ^Y^ ≦ 55°. This increase is due to the flocculation of the particles and the relationship between the flocculation and oil foaming behavior is discussed later.

OTFE dispersions at different *x*_MS_ (θ^Y^) and particle concentrations were vigorously shaken by a hand. An example of OTFE dispersions after aeration (3.0 vol.%) is shown in Figure 3, lower part. For pure *n*-heptane (*x*_MS_ = 0, θ^Y^ = 42°), the volume of the dispersion is unchanged from the original one upon aeration, implying no stable bubbles (hence foam) was formed. The case for pure methyl salicylate (*x*_MS_ = 1.0, θ^Y^ = 89°) also shows no practical change of the volume, but a white layer has appeared, which could be highly flocculated particles containing a relatively small number of air bubbles, as shown in Figure 5D. The flocculated particles with the air bubbles creams up due to a decrease in the apparent particle density, leading to the formation of the white layer. When the oil mixtures were used (0.12 ≦ *x*_MS_ ≦ 0.91, 45° ≦ θ^Y^ ≦ 76°), the dispersions show not only the formation of white layers but also an increase in the total volume, indicating air is incorporated into the samples as bubbles; oil foams are formed. It is found that the extent of volume increase is quite dependent on *x*_MS_ (and θ^Y^).

**Figure 4:**
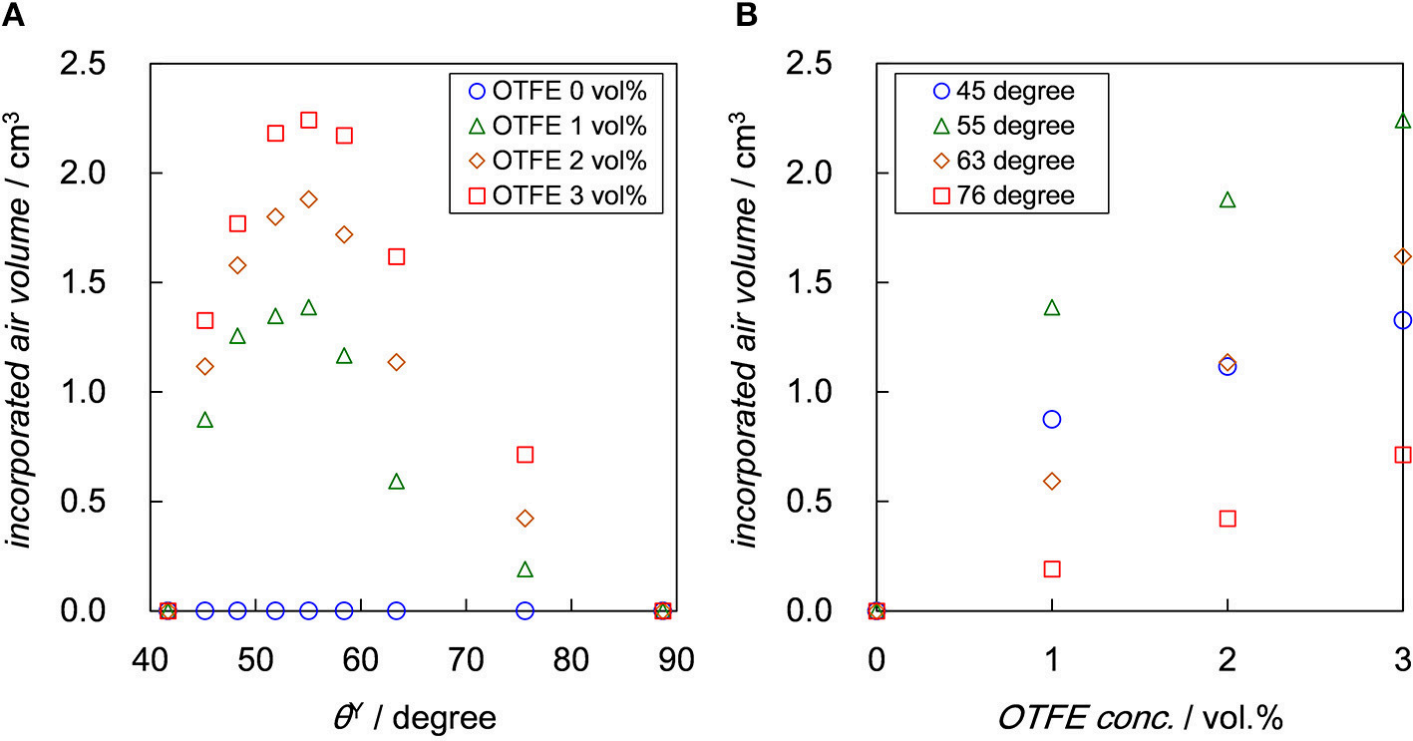
Air volume incorporated into the dispersion with different OTFE concentration after aeration plotted as a function of: **(A)** θ^Y^ and **(B)** OTFE concentration. Air volume measured after 1 month is plotted.

The air volume incorporated into the dispersions with different OTFE concentrations after aeration are dependent on both θ^Y^ and OTFE concentration, as shown in Figure 4. The total volume after aeration has unchanged with time, while a clear subphase appeared with time when θ^Y^ ≥ 45°, indicating creaming of bubbles. It should be stressed that the incorporated air volume has unchanged with time for all the θ^Y^ and the particle concentration for at least 3 months, suggesting the particle-stabilized bubbles and foams are stable against coalescence and disproportionation. On the dependence of the incorporated air volume on θ^Y^ (Figure 4A), there is a maximum of the incorporated air volume at θ^Y^ = 55°, irrespective of OTFE concentration; oil foams are efficiently formed when the contact angle is intermediate. For particle-stabilized foams prepared using water and ethanol mixture, there is also a maximum in foam height when the contact angle is between 75 and 85° (Sun and Gao, 2002). The existence of the maximum at the intermediate contact angle is not explained in terms of Δ*G* which increases with θ^Y^ approaching to 90° and is contradict with the behavior observed for the particle-stabilized aqueous foams (Binks and Horozov, 2005; Binks and Murakami, 2006; Binks et al., 2007). We discuss this unexpected oil foaming behavior later.

Figure 4B shows that the incorporated air volume monotonically increases with increasing OTFE concentration at a fixed θ^Y^. Binks and Tyiwua observed an increase in volume of particle-stabilized oil foams with increasing a particle concentration and pointed out the resemblance to surfactant foaming agents below the critical micelle concentration (Binks and Tyowua, 2013). It is assumed that during aeration bare bubbles are initially formed and if the bubbles are adsorbed by enough amount of particles during aeration, they remain as particle-stabilized bubbles after the cease of aeration. The size of bubbles stabilized by particles might be larger than the original bare bubble due to the limited coalescence (Arditty et al., 2003; Tcholakova et al., 2008). The increase in the particle concentration facilitate the bubble stabilization, which is driven by an increase in the potential total area to be covered by particles, leading to an increase in the incorporated air volume. However the drawback of the increase in the particle concentration is a decrease in the rate of energy dissipation, which is consumed by the formation bubble surfaces and the agitation of the total mass (Tsabet and Fradette, 2015). The air volume incorporated in oils is determined by a balance between these two factors; the air volume could initially increase and practically reach a plateau with increasing the particle concentration.

**Figure 5:**
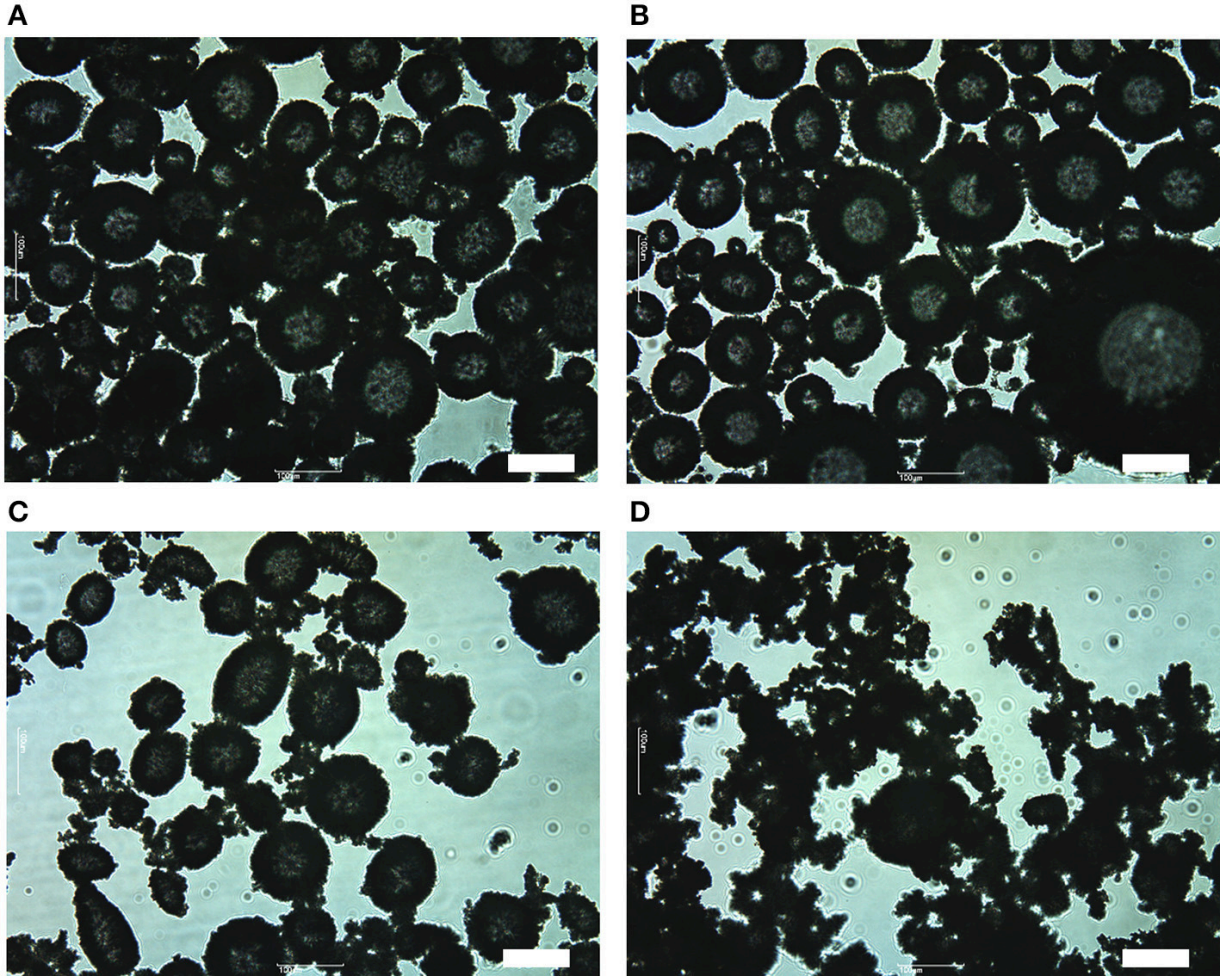
Optical micrographs of aerated OTFE dispersions with 3.0 vol.% of particle concentration at different θ^Y:^ θ^Y^ = **(A)** 48°, **(B)** 55°, **(C)** 63°, and **(D)** 76°. Scale bar = 100 μm.

Figure 5 shows optical micrographs of aerated OTFE dispersions with 3.0 vol.% of particle concentration. When θ^Y^ = 48 and 55°, a large amount of air bubbles are seen (Figures 5A,B). The typical bubble size ranges from tens micrometer to hundreds micrometer and the average size appears to be not dependent on θ^Y^. Most of the bubbles are deformed from a sphere, suggesting OTFE particles are jammed at the bubble surfaces (Subramaniam et al., 2005, 2006). When θ^Y^ = 63° (Figure 5C), less amount of bubbles are observed and some bubbles appear to be covered by the flocculated particles. There are only a few bubbles and a large amount of highly flocculated particles at a relatively high θ^Y^ (θ^Y^ = 76°, Figure 5D). The bubbles appear to be entrapped by the network of the flocculated particles. The extent of the deformation appears to become more severe with increasing θ^Y^; the particle networking might also retard the shape relaxation of the bubbles due to the air/oil surface tension.

### Oil foaming mechanism

**Figure 6:**
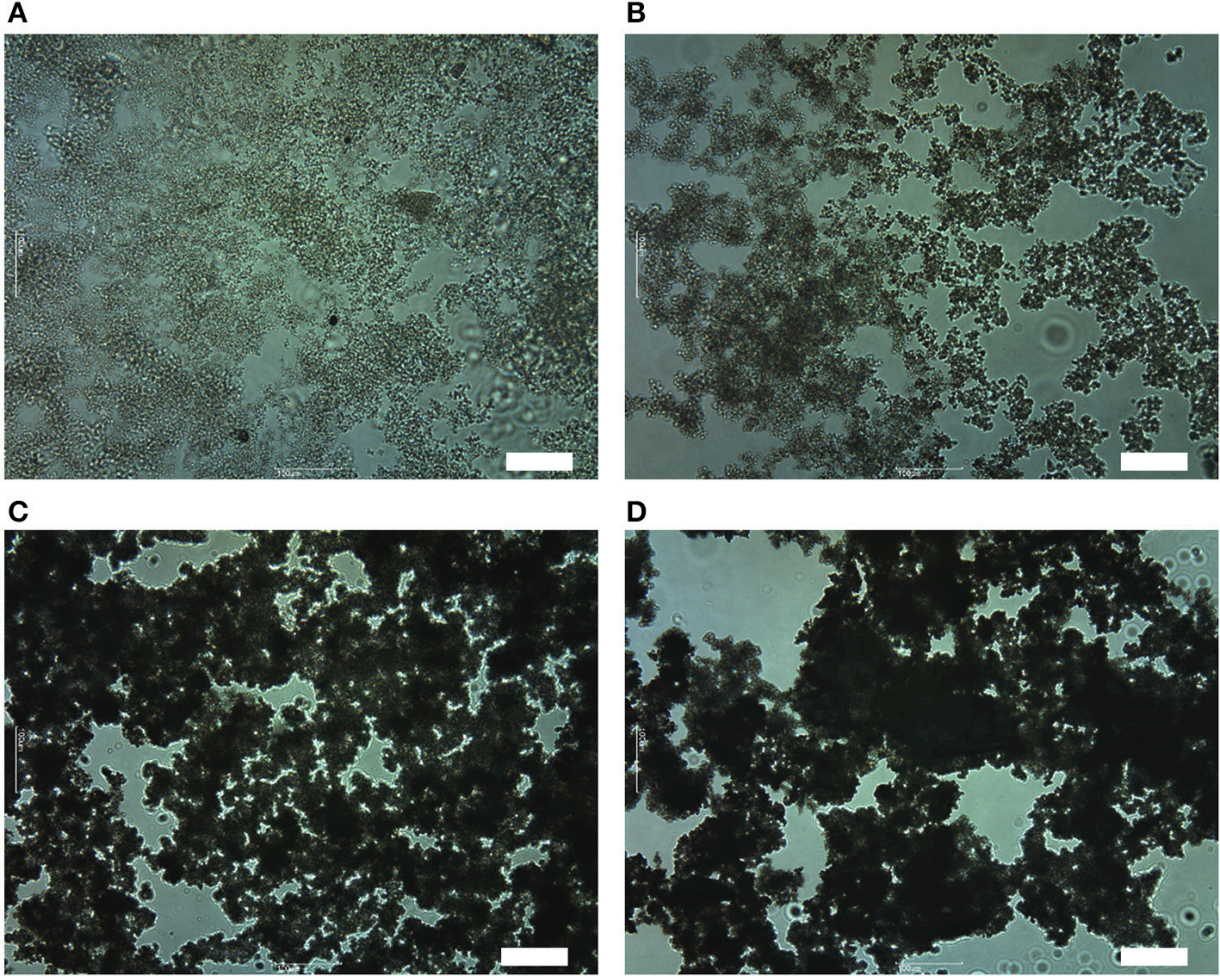
Optical micrographs of 1.0 vol.% dispersions of OTFE particles in oil mixtures at different θ^Y:^ θ^Y^ = **(A)** 42°, **(B)** 55°, **(C)** 63°, and **(D)** 76°. Scale bar = 100 μm.

To investigate the reasons why the foaming efficiency decreases when θ^Y^ > 55°. We have observed OTFE dispersions before aeration by an optical microscopy. When the contact angle is relatively low (for the case of pure *n*-heptane, θ^Y^ = 42°, Figure 6A), the particles appear reasonably dispersed. With increasing θ^Y^ (a mole fraction of methyl salicylate), however, OTFE particles are progressively flocculated in the oils Figures 6B–D). These findings are consistent with an increase in the apparent volume of the sedimented particles increasing θ^Y^ when θ^Y^ > 55°, as shown in Figure 3. It is thought that the particle packing in the sedimented layer progressively becomes loose with increasing the degree of the flocculation.

The flocculation is induced by either an increase in attractive interactions or a decrease in repulsive interactions. There would be no effective repulsive interactions between OTFE particles in oils, while it is reasonably assumed that the van der Waals interaction between OTFE particle surfaces is the origin of the attractive interaction. We have estimated the non-retarded Hamaker constant *A* for two OTFE particle surfaces interacting across an oil according to the Lifshitz theory (Israelachvili, 2011):

(2) $$\begin{array}{l} \text{A}=\frac{3}{4}kT{\left(\frac{\varepsilon_{p}-\varepsilon_{o}}{\varepsilon_{p}+\varepsilon_{o}}\right)}^{2}+\frac{3\text{h}\nu_{e}}{16\sqrt{2}}\frac{{\left(n_{\text{p}}^{2}-n_{\text{o}}^{2}\right)}^{2}}{{\left(n_{\text{p}}^{2}+n_{\text{o}}^{2}\right)}^{3/2}}, \end{array}$$

where ε_p_ and ε_o_ are the dielectric constant of OTFE particle and oil and *n*_p_ and *n*_o_ are the refractive index of OTFE particle and oil. *k, T, h*, and ν_e_ are the Boltzmann constant, the absolute temperature, the Plank constant and the main electronic absorption frequency in the UV, respectively. The Hamaker constants estimated for two OTFE particles in either *n*-heptane or methyl salicylate are shown in Table 1. The ε_p_ and *n*_p_ values were assumed to be same as those for PTFE (ε_p_ = 2.1 and *n*_p_ = 1.359) and the ν_e_ value was set to be 2.9 × 10^−15^ s^−1^, which is a typical value for aliphatic hydrocarbons and PTFE (Israelachvili, 2011). The Hamaker constant for methyl salicylate is about 40 times higher than that for *n*-heptane. It is expected that the Hamaker constant for mixtures of *n*-heptane and methyl salicylate increases with increasing *x*_MS_ (and θ^Y^). The increase in the Hamaker constant could be the reason for the increase in the degree of the flocculation.

**Table 1:** Dielectric constant, refractive index and Hamaker constants for two OTFE particles in oils at 293.15 K.

**Oil**	**ε_o_**	***n*_o_**	**A / 10^−21^ J**	**γ_al_ / mN m^−1^ (298.15 K)**	**θ^Y^/°**
*n*-heptane	1.92	1.38776*^*a*^*	0.23	19.66	42
Methyl salicylate	9.64	1.535	8.9	39.2	89
Cyclohexanone	16.1	1.507	7.3	34.57	–
Methyl benzoate	6.642*^*b*^*	1.5164	7.0	37.17	–
Benzaldehyde	17.85	1.5463	10.5	38.00	–

Referenced from Lide (2006), except

a *(Rodriguez et al., 1999)*.

b *Value at 302.7 K*.

The Hamaker constants were estimated for other three oils (cyclohexanone, methyl benzoate and benzaldehyde), whose surface tension is close to that for methyl salicylate and hence would show a relatively high contact angle. The Hamaker constants for the three oils are again much higher than that for *n*-heptane. By mixing an oil with a low surface tension and a high surface tension, the contact angle can be precisely controlled as shown the above, but the flocculation of OTFE particles is inevitable and the efficient oil foaming is not expected when a composition of an oil with a high surface tension in the oil mixture is relatively high.

Here we propose that the flocculation of the particles is closely related to the foaming behavior. One of the possible reasons for less foaming with highly flocculated particles could be a decrease in the effective particle concentration with increasing the degree of flocculation. In the field of mineral flotation, it is well-known that the rate of flotation is a first order to a bulk particle concentration;

(3) $$\begin{array}{l} \frac{\text{d}N}{\text{d}t}=-kN, \end{array}$$

where *N* is the number of floatable particles and *k* is the rate constant (Mao and Yoon, 1997; Arai et al., 2009). Upon aeration, as stated in the previous section (the dependence of OTFE concentration on the incorporated air volume), bare bubbles could be initially formed and the bubbles become stable against coalescence if the bubbles are enoughly covered by the particles during aeration. The finding that the incorporated air volume increases with increasing the bulk OTFE concentration has suggested that the efficiency of the coverage could increase with increasing the particle concentration around bubbles. Not well-covered bubbles could be formed at a low effective particle concentration for flocculated particles and they are prone to coalesce during aeration, eventually less amount of foam is produced compared to the more dispersed particles. On one hand, the low degree of flocculation might be advantageous to the formation of oil foams. The degree of the flocculation could monotonically increase with increasing the contact angle in this study (see Figure 6). The maximum foaming at the intermediate contact angle (55°) might be associated with the particle adsorption energy but also the moderately flocculated particles that can retard drainage and improve the stability of oil foams. Another reason might be that flocculated particles are so large that they are more slowly transferred to an air/oil surface than the deflocculated ones.

## Conclusion

Foaming of oils using a particulate material (particles of oligomer of tetrafluoroethylene) was carried out by controlling the particle contact angle against air/oil surfaces and the particle concentration. OTFE particles were properly dispersed in the oils to eliminate the oil-repellency character due to the metastable Cassie-Baxter state. The particle contact angle was controlled by changing a composition of mixtures of two oils with different surface tensions. Oil foaming efficiency shows a maximum at an intermediate contact angle (55°), which is contradict to the findings for particle-stabilized aqueous foams. The reason for the poor oil foaming when the contact angle is relatively high could be a decrease in the effective particle concentration with increasing the degree of flocculation. Less covered bubbles are formed for the flocculated particles and they could be less stable against coalescence. If particles are well dispersed even at a high contact angle, the efficient formation of particle-stabilized oil foams is expected. It is worth using sterically stabilized particles, such as particles with a hairy polymer layer solvated with oil, to test this idea.

## Author contributions

RM designed the study, and wrote the initial draft of the manuscript. MY and AB contributed to analysis and interpretation of data, and assisted in the preparation of the manuscript. SK and MO have contributed to data collection and interpretation.

### Conflict of interest statement

The authors declare that the research was conducted in the absence of any commercial or financial relationships that could be construed as a potential conflict of interest.
